# The Effect of Disease-Modifying Drugs on Brain Atrophy in Relapsing-Remitting Multiple Sclerosis: A Meta-Analysis

**DOI:** 10.1371/journal.pone.0149685

**Published:** 2016-03-16

**Authors:** Pierre Branger, Jean-Jacques Parienti, Maria Pia Sormani, Gilles Defer

**Affiliations:** 1 Department of Neurology, University Hospital of Caen, Caen, France; 2 Biostatistics and clinical research unit, University Hospital of Caen, Caen, France; 3 Biostatistics Unit, Department of Health Sciences, University of Genoa, Genoa, Italy; National Institutes of Health, UNITED STATES

## Abstract

**Background:**

The quantification of brain atrophy in relapsing-remitting multiple sclerosis (RRMS) may serve as a marker of disease progression and treatment response. We compared the association between first-line (FL) or second-line (SL) disease-modifying drugs (DMDs) and brain volume changes over time in RRMS.

**Materials and Methods:**

We reviewed clinical trials in RRMS between January 1, 1995 and June 1, 2014 that assessed the effect of DMDs and reported data on brain atrophy in Medline, Embase, the Cochrane database and meeting abstracts. First, we designed a meta-analysis to directly compare the percentage brain volume change (PBVC) between FLDMDs and SLDMDs at 24 months. Second, we conducted an observational and longitudinal linear regression analysis of a 48-month follow-up period. Sensitivity analyses considering PBVC between 12 and 48 months were also performed.

**Results:**

Among the 272 studies identified, 117 were analyzed and 35 (18,140 patients) were included in the analysis. Based on the meta-analysis, atrophy was greater for the use of an FLDMD than that of an SLDMD at 24 months (primary endpoint mean difference, -0.86; 95% confidence interval: -1.57–-0.15; P = 0.02). Based on the linear regression analysis, the annual PBVC significantly differed between SLDMDs and placebo (-0.27%/y and -0.50%/y, respectively, P = 0.046) but not between FLDMDs (-0.33%/y) and placebo (P = 0.11) or between FLDMDs and SLDMDs (P = 0.49). Based on sensitivity analysis, the annual PBVC was reduced for SLDMDs compared with placebo (-0.14%/y and -0.56%/y, respectively, P<0.001) and FLDMDs (-0.46%/y, P<0.005), but no difference was detected between FLDMDs and placebo (P = 0.12).

**Conclusions:**

SLDMDs were associated with reduced PBVC slope over time in RRMS, regardless of the period considered. These results provide new insights into the mechanisms underlying atrophy progression in RRMS.

## Introduction

The development of disease-modifying drugs (DMDs) for multiple sclerosis (MS) has been based on their observed effects on clinical outcomes, including the rate of relapse and the accumulation of permanent disability [1,2]. Aside from these clinical parameters, changes in brain lesion burden have been commonly used to monitor the in vivo effects of DMDs based on conventional MRI, which serves as a potential surrogate marker in MS trials. However, the use of this measure has been disputed because of the poor correlation between MRI-based measures of inflammatory activity and relapse and disability progression [3,4].

Although inflammation and focal demyelination are the pathological hallmarks of MS, the occurrence of brain atrophy is currently a classical characteristic of cross-sectional and longitudinal imaging studies beginning at the earliest stage of the disease and proceeding throughout the disease course [5]. Because brain atrophy represents the net effect of primary disease-related pathophysiological processes, including demyelination, axonal loss and neurodegeneration, quantifying brain volume changes may represent a promising MRI outcome measure to evaluate the expected or unexpected (neuroprotective) effects of DMDs [6,7].

Nearly all phase II and III clinical trials concerning first-line (FL) or second-line (SL) DMD previously included brain atrophy as outcome measure, we were interested in determining the manner in which SLDMDs and FLDMDs modified brain atrophy progression over time. First, we performed a meta-analysis to compare the effect of FLDMD, SLDMD and placebo on atrophy. Second, we conducted an observational and longitudinal linear regression analyses to evaluate the potential association between DMDs and brain volume changes over time.

## Materials and Methods

### Search strategy and selection criteria

This study has adopted the Preferred Reporting Items for Systematic Reviews and Meta-Analyses (PRISMA) guidelines (S1 PRISMA Checklist) [8]. We collected all reports fulfilling the following selection criteria: trials of relapsing-remitting MS (RRMS) assessing the efficacy of a DMD and reporting cerebral atrophy data. The literature search was performed in MEDLINE (PubMed), EMBASE, and CENTRAL (the Cochrane Library) using terms for the disease name (“multiple sclerosis”), “atrophy” and DMDs ("glatiramer acetate" or "interferon" or "teriflunomide" or "BG-12" or "laquinimod" or "natalizumab" or "fingolimod" or "alemtuzumab" or "daclizumab" or "ocrelizumab") from January 1, 1995, to June 1, 2014. The complete search algorithm that was used in MEDLINE search is avalaible in the S1 Text. We also included the phase II and III trials of each drug, which were occasionally collected directly from the authors if they were not published, and posters or abstracts from scientific meetings. The abstracts were independently screened for article selection, and full-length articles were examined if relevant information could not be ascertained from the abstracts. The studies were selected according to inclusion criteria, and we excluded studies that did not report atrophy results and excluded duplicate records between databases.

### Data extraction

We reviewed the full text of all selected studies and extracted data from all studies containing PBVC, the brain volume or the brain fraction at each imaging time point among RRMS patients from baseline to up to 48 months. For each study, we collected the design of the study (randomized, placebo-controlled and active-controlled), the technique used to measure PBVC, and the name and dose of the DMD used. Then, for each arm of each study (an arm was a group of patients treated with the same DMD at the same dose), we collected the sex ratio, age, EDSS, previous annual relapse rate, duration of disease progression since the first symptoms or diagnosis, number of randomized patients and number of patients at each imaging time point. When clinical data related to the atrophy measurement were missing from the article, we referred to the pivotal trial article. If the number of patients was not indicated at one time point, we recorded the number of initially randomized patients as the appropriate value. Glatiramer acetate, interferon, teriflunomide, BG-12 and laquinimod were classified as FLDMDs, and natalizumab, fingolimod and alemtuzumab were classified as SLDMDs according to the health authorities’ recommendations. Daclizumab was considered an SLDMD based on the previous health authorities’ recommendations for immunosuppression in organ transplant patients.

### Endpoints

The endpoint was PBVC from baseline to up to 48 months and was compared between FLDMD, SLDMD and placebo. In studies using the SIENA method, PBVC was automatically calculated and reported by authors. Some trials performed repeated measurements, whereas others performed only one PBVC measurement. For other methods of atrophy measurement, the brain volume or brain fraction at each time point was reported, and the authors calculated the percentage brain volume or fraction change from baseline, which we considered the PBVC. If the PBVC was not calculated by the authors, we estimated it using the following formula: (brain volume or fraction at the given time point–brain volume or fraction at baseline) / brain volume or fraction at baseline. A negative result represented a decrease in brain volume, indicating atrophy progression.

### Statistical methods

The baseline characteristics for each qualified study and DMD are presented as the means and standard deviations (SD) when available. To examine the association between FLDMD, SLDMD and placebo and PBVC over time, we performed two statistical approaches.

First, we performed a meta-analysis of exclusively those studies that compared the PBVC between an FLDMD and placebo, an SLDMD and placebo or an FLDMD and SLDMD at 12 or 24 months at the recommended doses using Review Manager (RevMan, Version 5.3, Copenhagen: The Nordic Cochrane Centre, The Cochrane Collaboration, 2014). Continuous outcomes were expressed as the means and SD. If the SD was missing, we used a weighted average of the SD reported in other studies of the same class of DMD and measurement period. If the I² statistic heterogeneity measure exceeded 50%, we used a random effects model. In each study included in the meta-analysis, a predefined 7-point quality control was used to address for biases [9] and funnel plot was performed to evaluate the publication bias.

Second, we performed a linear regression analysis using a Generalized Estimating Equations model, adjusting for time-trend and group, which was appropriate for repeated longitudinal data. Formally, we tested the interaction between each pair-wise DMD and time to explore the potential differences in brain atrophy over time. We weighted each brain volume change according to the number of patients examined at each time point. In the overall analysis over the 48-month follow-up period, we included all studies and all PBVC values. In a secondary analysis performed between 12 and 48 months to correct for any possible pseudoatrophy effects [10], we included exclusively those studies that reported the PBVC at 12 months and at least one other time point thereafter. For complementary sensitivity analyses, we evaluated only randomized studies that used the current clinically recommended dose when 2 doses were available in the same study and used the best-validated methods of atrophy measurement (SIENA and BPF) [11,12].

A P-value less than 0.05 was considered to denote statistical significance. No adjustment was made for multiple comparisons. We used the SAS v9.4 software (SAS Institute, Inc., Cary, NC, USA) for statistical analysis.

## Results

### Number of studies

**Fig 1:**
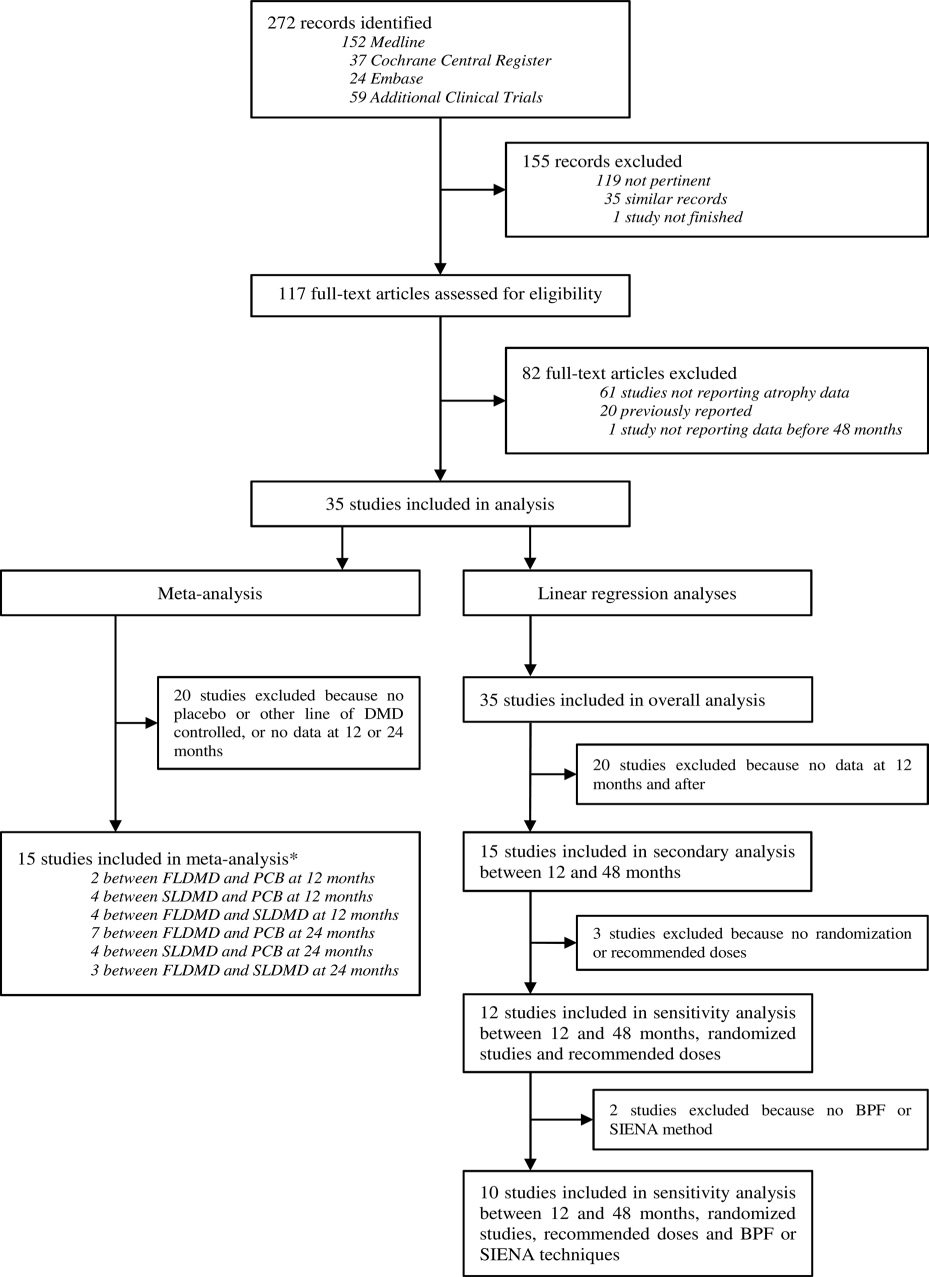
Flow chart of systematic review. *One study could be included at 12 and 24 months. DMD: Disease Modifying Drug; FLDMD: First-line DMD; SLDMD: Second-line DMD; PCB: Placebo; BPF: Brain Parenchymal Fraction; SIENA: Structural Image Evaluation, using Normalisation, of Atrophy.

We identified 272 studies between January 1, 1995, and June 1, 2014, including 152 in MEDLINE, 37 in COCHRANE, 24 in EMBASE and we included an additional 59 clinical trials (Fig 1). Based on a screen of the abstracts, 155 studies were excluded because of impertinent or duplicate records. The full texts of 117 studies were analyzed. Sixty-one studies were excluded due to the lack of PBVC data, 20 were excluded because the data had been previously reported and 1 was excluded because it did not report data before 48 months from baseline. We analyzed 35 studies representing 71 arms (some studies included 3 comparative arms, and others included only one arm in which the brain volume was monitored). In the meta-analysis, we included 15 head-to-head studies of the PBVC at 12 or 24 months. Risk of bias is summarized in S1 and S2 Figs. In the observational and longitudinal linear regression analyses, all 35 studies were included in the overall analysis, and between 10 and 15 studies were included in the secondary analyses according to the criteria described above.

### Characteristics of the studies and the population

The 35 included studies are presented in S1 Table. Table 1 summarizes the primary characteristics of the studies, and Table 2 shows the baseline characteristics of the FLDMD, SLDMD and placebo arms. These studies represented 18,140 patients, 51% of whom were treated with an FLDMD, 27% of whom were treated with an SLDMD, and 22% of whom received placebo. The primary baseline characteristics of the patients (age, disease duration, EDSS, and annualized relapse rate) did not differ between the FLDMD and SLDMD groups.

**Table 1:** Primary characteristics of the 35 included studies.

	All studies, *n = 35*
Design^a^	
Placebo-controlled, n (%)	16 (46)
Active-controlled, n (%)	11 (31)
Different doses, n (%)	14 (40)
Not randomized, n (%)	10 (29)
Number of arms	
1, n (%)	7 (20)
2, n (%)	14 (40)
3, n (%)	14 (40)
Type	
Phase 2, n (%)	3 (9)
Phase 3, n (%)	17 (49)
Others, n (%)	15 (43)
Technique	
BPF, n (%)	9 (26)
SIENA, n (%)	17 (49)
Other, n (%)	9 (26)

^a^One study could be in two designs.

BPF: Brain Parenchymal Fraction; SIENA: Structural Image Evaluation, using Normalisation, of Atrophy.

**Table 2:** Baseline characteristics of FLDMD, SLDMD and placebo arms.

	FLDMD							SLDMD					PCB	All DMD
	GA, *n = 6*	IFN, *n = 19*	GA or IFN^a^, *n = 2*	TER, *n = 2*	LAQ, *n = 2*	BG-12, *n = 2*	All, *n = 33*	NTZ, *n = 5*	FIN, *n = 8*	ALM, *n = 5*	DAC, *n = 2*	All, *n = 20*	PCB, *n = 18*	All, *n = 71*
Sex ratio, f/m														
Mean	2.50	2.29	2.19	2.37	2.20	3.26	2.38	2.55	2.49	1.96	1.9	2.32	2.64	2.42
SD	0.26	0.30	0.02	0.08	0.30	0.09	0.34	0.28	0.51	0.20	0.13	0.48	0.70	0.49
Age, years														
Mean	36.06	36.35	32.88	37.60	38.06	38.40	36.61	35.35	37.88	33.76	35.25	36.30	37.52	36.72
SD	0.66	1.95	3.01	0.20	0.94	0.10	1.73	0.91	1.88	1.17	0.05	2.33	1.59	1.94
EDSS, no														
Mean	2.22	2.44	2.31	2.68	2.64	2.30	2.42	2.47	2.36	2.34	2.75	2.40	2.43	2.42
SD	0.11	0.37	0.30	0.01	0.05	0.00	0.31	0.45	0.12	0.36	0.05	0.29	0.29	0.30
ARR, no														
Mean	1.49	1.54	0.97	1.35	1.24	1.30	1.46	2.22	1.47	1.72	1.35	1.53	1.35	1.46
SD	0.08	0.12	0.45	0.05	0.05	0.00	0.17	0.14	0.06	0.07	0.05	0.16	0.09	0.16
Duration, years														
Mean	4.45	6.00	7.60	8.75	7.77	8.34	6.16	5.60	8.67	3.11	3,0	6.40	7.73	6.57
SD	1.58	1.63	2.51	0.05	1.04	0.35	1.98	1.58	1.30	1.36	0.00	2.84	1.85	2.31
Patients, no	2 108	4 948	104	725	984	360	9 229	731	2 624	1 194	417	4 966	3 945	18 140

^a^2 studies used GA or IFN in the same arm.

DMD: Disease Modifying Drug; FLDMD: First-line DMD; SLDMD: Second-line DMD; PCB: Placebo; GA: Glatiramer Acetate; IFN: Interferon; TER: Terifluomide; LAQ: Laquinimod; BG-12: dimethyl fumarate; NTZ: Natalizumab; FIN: Fingolimod; ALM: Alemtuzumab; DAC: Daclizumab; n: number of arms included in the analyses; EDSS: Expended Disability Status Scale; ARR: Annual Relapse Rate; Duration: disease duration between first relapse or diagnostic; SD: Standard Deviation.

### Meta-analysis

**Fig 2:**
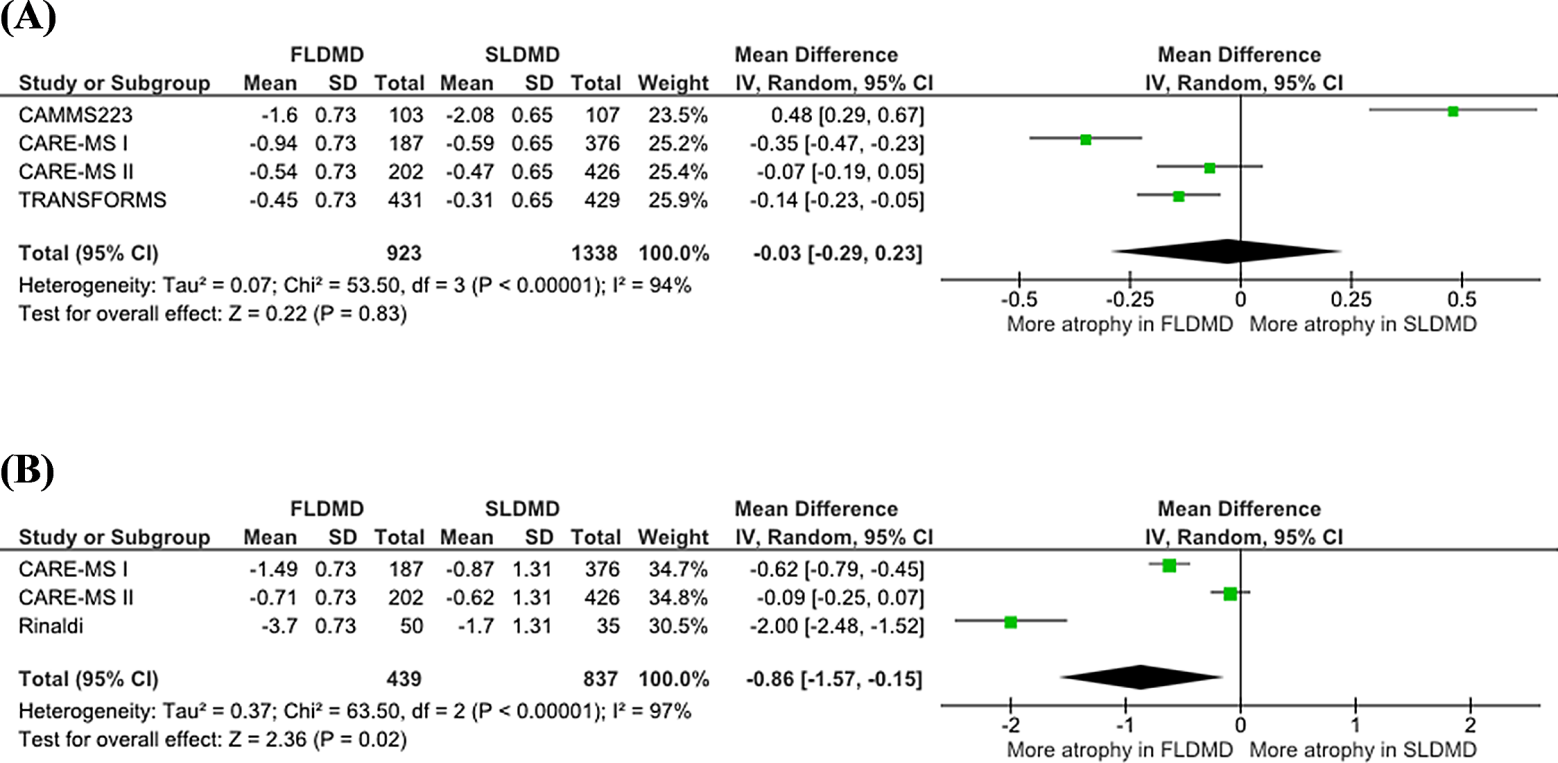
**Forest plot of comparison between FLDMD and SLDMD at 12 months (A) and 24 months (B).** DMD: Disease Modifying Drug; FLDMD: First-line DMD; SLDMD: Second-line DMD; SD: Standard Deviation.

At 12 months, no significant differences were detected between SLDMDs and placebo (primary endpoint mean difference, 0.05; 95% confidence interval (CI): -0.14–0.24; P = 0.62; Figure A in S3 Fig), between FLDMDs and placebo (0.02; 95% CI: -0.26–0.30; P = 0.87; Figure B in S3 Fig) or between FLDMDs and SLDMDs (-0.03; 95% CI: -0.29–0.23; P = 0.83; Fig 2A).

At 24 months, greater atrophy was detected in the placebo group than in the SLDMD group (0.85; 95% CI: 0.21–1.48; P = 0.009; Figure C in S3 Fig) and the FLDMD group (0.30; 95% CI: 0.11–0.48; P = 0.002; Figure D in S3 Fig). The comparison between FLDMD and SLDMD revealed significantly greater atrophy in the FLDMD group than the SLDMD group (−0.86; 95% CI: −1.57–−0.15; P = 0.02; Fig 2B). Publication bias is avalaible in S4 Fig.

### Observational and longitudinal linear regression analyses

#### Overall analysis

**Fig 3:**
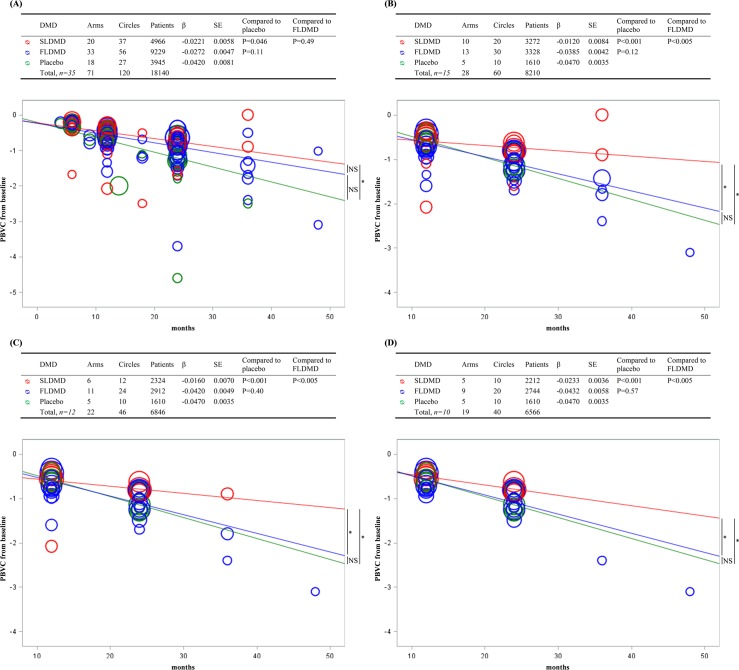
**Evolution of the percentage brain volume change up to 48 months (A), from 12 to 48 months (B), from 12 to 48 months using only randomized studies and recommended doses (C) and from 12 to 48 months using only randomized studies, recommended doses and BPF or SIENA techniques (D).** *, significant, see p-value in the associated table. DMD: Disease Modifying Drugs; FLDMD: First-line DMD; SLDMD: Second-line DMD; β: Coefficient of monthly PBVC slope; SE: Standard Error; PBVC: Percentage Brain Volume Change; NS: Not significant. Each Circle represents the percentage brain volume change from baseline, in one group of patients treated in the same study by the same treatment. Circle is proportional to the sample size of the studied arms.

The PBVC slope was negative in all groups studied between months 0 and 48. Atrophy was more pronounced in the placebo group, with an estimated annual PBVC slope of -0.50%/y. The estimated annual PBVC was -0.33%/y for patients who received an FLDMD and -0.27%/y for those who received an SLDMD (Fig 3A). A significant difference was detected between the SLDMD and placebo groups (P = 0.046) but not between the FLDMD and SLDMD groups (P = 0.49) or between the FLDMD and placebo groups (P = 0.11).

#### Secondary analyses

Fifteen studies were analyzed that included data between 12 and 48 months (8210 patients) (Fig 3B). The annual PBVC slope was reduced for SLDMDs compared with placebo (-0.14%/y and -0.56%/y, respectively, P<0.001) and FLDMDs (-0.46%/y, P<0.005), but no difference was detected between FLDMDs and placebo (P = 0.12).

Other complementary sensitivity analyses, which evaluated only randomized trials that used the currently recommended doses (12 studies, Fig 3C) and the BPF or SIENA method (10 studies, Fig 3D), confirmed the greater reduction in atrophy among the patients who received an SLDMD compared with those who received an FLDMD or placebo (P<0.005 and P<0.001, respectively, in both analyses). No difference in atrophy was detected between FLDMDs and placebo based on each analysis (P = 0.40 and P = 0.47, respectively).

## Discussion

The present study of all clinical trials that included the effect of an FLDMD or SLDMD on brain atrophy as an outcome measure showed that in patients with RRMS, the use of an SLDMD significantly decreased the slope of PBVC compared with placebo over a period of 48 months. In contrast, we did not detect any differences in the PBVC slope between FLDMD and placebo based on either the primary and secondary analyses. The effect of SLDMDs on PBVC was significantly greater than that of FLDMDs at 24 months based on the meta-analysis and between 12 and 48 months based on the observational longitudinal linear regression analyses, including sensitivity analyses of randomized clinical trials that used the recommended doses and validated methods of atrophy measurement. The observed superior effect of SLDMDs to FLDMDs on the PBVC over time is intriguing.

Several mechanisms may underlie the observed difference in the PBVC slope between the use of an FLDMD and an SLDMD. The powerful and sustained anti-inflammatory effect may be considered the primary factor that explains the observed difference in PBVC progression between SLDMDs and FLDMDs and placebo. In all head-to-head studies, SLDMDs displayed a larger clinical effect on relapse and MRI activity than interferon or glatiramer acetate [13–15]. However, other in vitro or in vivo studies in the field of neuroprotection suggested that some SLDMDs, such as fingolimod, may display additional pharmacological properties via direct interactions with glial cells and neurons [16]. Other specific potentially protective mechanisms that are not directly related to anti-inflammatory effects have been suggested for monoclonal antibodies [17,18]. However, similar interesting mechanisms in the field of neuroprotection have been described for some FLDMDs [19,20]. Therefore, the exact mechanisms that affect PBVC over time remain to be elucidated, even if the larger anti-inflammatory effect of SLDMDs may ultimately be sufficient to explain the results of our study based on considering the convincing data linking the neurodegenerative process to the initial inflammatory process in MS [21,22].

Evaluating the prevention of brain atrophy as a reliable outcome measure of drug effectiveness is currently an active topic in MS research [6,23,7]. Previously, assessment of drug effectiveness mainly belongs to changes for T2 lesion load, gadolinium-enhancing lesions and T1 black hole lesions. However, depending of the disability outcome criteria, method used to measure lesions and time endpoint of the study, weak or no correlation were observed with one or all these MRI criteria [24–27]. On the contrary, brain atrophy may represent a promising MRI outcome measure considering pathophysiological processes including demyelination, axonal loss and neurodegeneration. Recently, Sormani et al. published a meta-analysis [28] exploring the relationship between the treatment effect size on brain atrophy and the treatment effect size on disability progression. In that study, the authors demonstrated that the treatment effect on disability progression correlated with the treatment effect on both brain atrophy and active MRI lesions. Despite a weak correlation coefficient between the treatment effect on brain atrophy and that on MRI lesions (R = 0.2), the variable “atrophy” in the multivariate weighted regression independently correlated with the treatment effect on disability progression, suggesting that the effect on brain atrophy contributed to that on the focal lesions to slow disability progression, which may be clinically relevant. Although our goal and methods were different from this previous study, our findings examined another important aspect of brain atrophy measurement over time, the possible difference in the treatment effect on PBVC between different types of DMDs. In their study, Sormani et al. did not classify the DMDs, and the duration of evaluation was limited to 2 years after randomization according to the endpoint disability progression over the same period. By extending the assessment period to 48 months and separately examining the post-pseudoatrophy period (12–48 months), we added complementary data on the duration and level of the effect of certain DMDs on PBVC over time. Accordingly, our findings suggest that the larger effect of SLDMDs on the rate of brain atrophy may predict their lower risk of disability progression than FLDMDs. However, this result remains to be formally demonstrated at the trial and individual levels for each SLDMD included in this analysis.

Our study contains some limitations. First, the drugs included in the DMD classes differed in terms of mode of action but the classification method used was based on a large professional consensus in the literature [29,30] and the recommendations of European health authorities for all DMDs, except for daclizumab for which recommendations have yet to be provided by these regulatory agencies. We decided to classify daclizumab into the SLDMD group based on its pharmacological profile and its previous use for the prevention of allograft rejection during renal transplantation [18]. Second, the methods used to assess PBVC differed between the studies included. However, in the meta-analysis, we used random effect models which account for this heterogeneity in the precision of the DMD effect size assessments and in the longitudinal analysis, we conducted subgroup analyses including only homogeneous techniques of atrophy measurement. BPF and SIENA are the most frequently used methods to measure brain volume in clinical practice and in MS trials of DMDs [31]. In a larger clinical trial dataset, blinded analysis by separate MRI reading centers using either SIENA or BPF measurements showed similar trends with a highly correlation at baseline and for brain volume change [32]. Third, one study [33] in the meta-analysis had PBVC of -3.7% in the FLDMD arm, appearing to be very different from other studies, and may represent a potential bias. Fourth, the complementary longitudinal analysis of PBVC over time included non-randomized single-arm studies and dissociated the arms of the randomized studies. Therefore, the results of the slope comparison between the DMD groups, even if they are more powerful than those of the meta-analysis, should be interpreted with caution. Nevertheless, several subgroup analyses were conducted including only randomized clinical trials, recommended doses and homogeneous techniques of atrophy measurement to assess the robustness of our conclusions [28,34]. Finally, the results of both, the meta-analysis and the linear regression analyses, consistently revealed a superiority of SLDMDs to FLDMDs regarding PBVC (see Fig 3B–3D).

Despite the correlation between some DMDs and the slowing of brain atrophy, we cannot confirm a relationship for any specific treatment between its treatment effect, the progression of atrophy and the clinical status at the individual level. Future studies with a primary objective of determining the relationship between atrophy and progression of motor or cognitive impairment are needed. In addition, this study is limited to the RR phase of MS, and these results cannot be applied to the progressive phase of MS. Moreover, our analysis was limited to 48 months of follow-up and could not be extended to a longer period due to the lack of studies beyond this period.

Our findings may have important clinical implications regarding therapeutic strategies for patients with RRMS. In fact, individual SLDMDs have previously displayed larger effects on the annual relapse rate and/or disability accumulation in patients than classical FLDMDs based on head-to-head comparisons [13–15]. If brain atrophy is considered a highly clinically relevant marker of disease progression that predicts the progression of motor and/or cognitive disability [35–38], our findings suggest that the use of an SLDMD in the therapeutic strategy should be considered as soon as possible according to the McDonald criteria [39] if MRI follow-up reveals any brain volume change. For patients who must shift from an FLDMD to an SLDMD according to the recommendations of health authorities, our data suggest that this SLDMD should be maintained as long as possible, even if clinical and radiological progression is lacking, because a disease-free status does not ensure the absence of neurodegeneration [40]. In this context, the recent proposition of Kappos et al. to include brain volume loss in a revised measure of MS disease-activity freedom provides novel insight into the assessment of the overall effects of DMDs on MS disease and guides the therapeutic strategy for RRMS patients [41]. Clearly, this should need a large diffusion of atrophy measurement as a routine radiological parameter in MRI follow-up.

In the near future, the availability of new DMDs that may exhibit neuroprotective effects could emerge from the private and/or academic manufacturing drug pipeline, likely leading to the use of brain atrophy measurement as a major surrogate marker of treatment efficacy for all MS forms. Our findings suggest that the PBVC should be monitored over a sufficient period of time. Regardless of the potential clinical value of this evaluation, this measure will likely be used in addition to the global assessment of disease progression to ensure that the reduction in the brain atrophy slope is formally clinically relevant.

## Supporting Information

S1 PRISMA Checklist PRISMA Checklist. (PDF)

S1 Fig Risk of bias summary: review authors' judgements about each risk of bias item for each included study. (PDF)

S2 Fig Risk of bias graph: review authors' judgements about each risk of bias item presented as percentages across all included studies. (PDF)

S3 Fig Forest plot of comparison between SLDMD and placebo at 12 months (A), FLDMD and placebo at 12 months (B), SLDMD and placebo at 24 months (C), FLDMD and placebo at 24 months (D).
(PDF)

S4 Fig Funnel plots of the included studies: FLDMD vs placebo at 12 monts (A), SLDMD vs placebo at 12 monts (B), FLDMD vs SLDMD at 12 months (C), FLDMD vs placebo at 24 monts (D), SLDMD vs placebo at 24 monts (E) and FLDMD vs SLDMD at 24 months (F).
(PDF)

S1 Table Baseline characteristics of included trials. (PDF)

S1 Text MEDLINE search algorithm. (PDF)
