# Supplementary material for: The Effect of Disease-Modifying Drugs on Brain Atrophy in Relapsing-Remitting Multiple Sclerosis: A Meta-Analysis
Source: PLoS One. 2016 Mar 16;11(3):e0149685. doi: 10.1371/journal.pone.0149685 (PMC4794160; doi:10.1371/journal.pone.0149685)

S2 Fig

Risk of bias graph: review authors' judgements about each risk of bias item presented as percentages across all included studies.

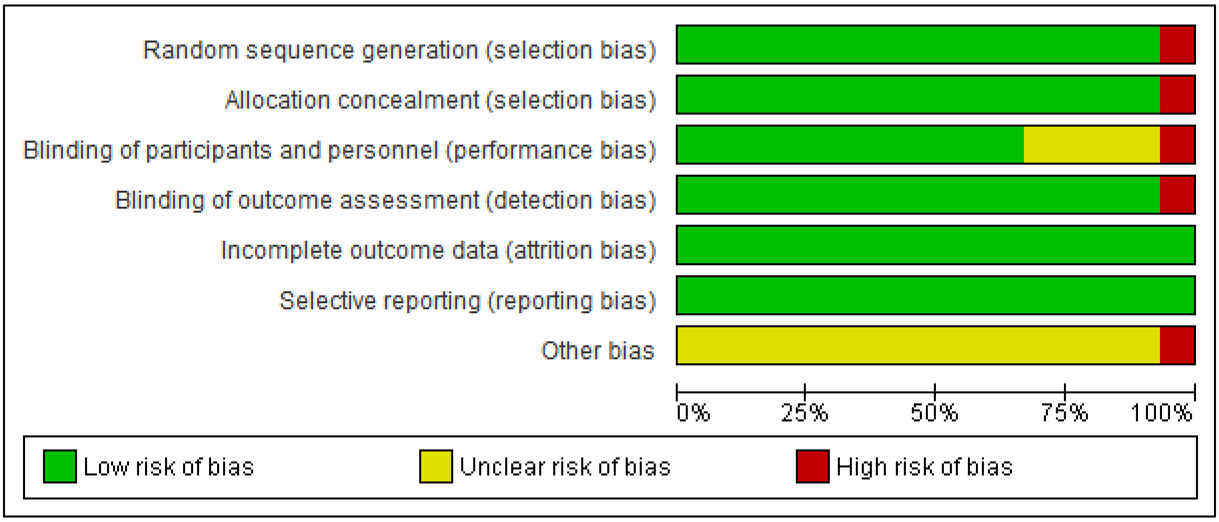

Supplement: S2 Fig — (PDF) [file pone.0149685.s003.pdf]
