# Supplementary material for: The Effect of Disease-Modifying Drugs on Brain Atrophy in Relapsing-Remitting Multiple Sclerosis: A Meta-Analysis
Source: PLoS One. 2016 Mar 16;11(3):e0149685. doi: 10.1371/journal.pone.0149685 (PMC4794160; doi:10.1371/journal.pone.0149685)
Supplement: S3 Fig — Forest plot of comparison between SLDMD and placebo at 12 months (A), FLDMD and placebo at 12 months (B), SLDMD and placebo at 24 months (C), FLDMD and placebo at 24 months (D). (PDF) [file pone.0149685.s004.pdf]

S3 Fig

Forest plot of comparison between SLDMD and placebo at 12 months (A), FLDMD and placebo at 12 months (B), SLDMD and placebo at 24 months (C), FLDMD and placebo at 24 months (D).

(A)

	SLDMD			PCB				Mean Difference
Study or Subgroup	Mean	SD	Total	Mean	SD	Total	Weight	IV, Random, 95% CI
AFFIRM	-0.56	0.97	606	-0.4	1.02	296	25.7%	-0.16 [-0.30, -0.02]
FREEDOMS	-0.5	1.05	383	-0.65	1.05	358	25.1%	0.15 [-0.00, 0.30]
FREEDOMS II	-0.38	0.97	300	-0.63	1.05	393	25.1%	0.25 [0.10, 0.40]
SELECT	-0.79	0.83	198	-0.74	0.9	194	24.0%	-0.05 [-0.22, 0.12]
**Total (95% CI)**			**1487**			**1241**	**100.0%**	**0.05 [-0.14, 0.24]**

Heterogeneity: Tau² = 0.03; Chi² = 18.39, df = 3 (P = 0.0004); I² = 84%
Test for overall effect: Z = 0.49 (P = 0.62)

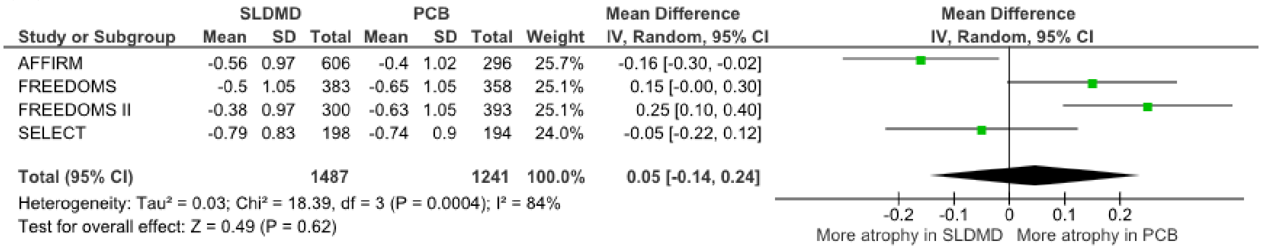

(B)

	FLDMD			PCB				Mean Difference
Study or Subgroup	Mean	SD	Total	Mean	SD	Total	Weight	IV, Random, 95% CI
BRAVO	-0.33	0.73	434	-0.58	1.02	225	36.1%	0.25 [0.10, 0.40]
BRAVO	-0.72	0.73	447	-0.58	1.02	225	36.1%	-0.14 [-0.29, 0.01]
MSCRG	-0.76	0.73	68	-0.7	1.02	72	27.9%	-0.06 [-0.35, 0.23]
**Total (95% CI)**			**949**			**522**	**100.0%**	**0.02 [-0.26, 0.30]**

Heterogeneity: Tau² = 0.05; Chi² = 13.55, df = 2 (P = 0.001); I² = 85%
Test for overall effect: Z = 0.16 (P = 0.87)

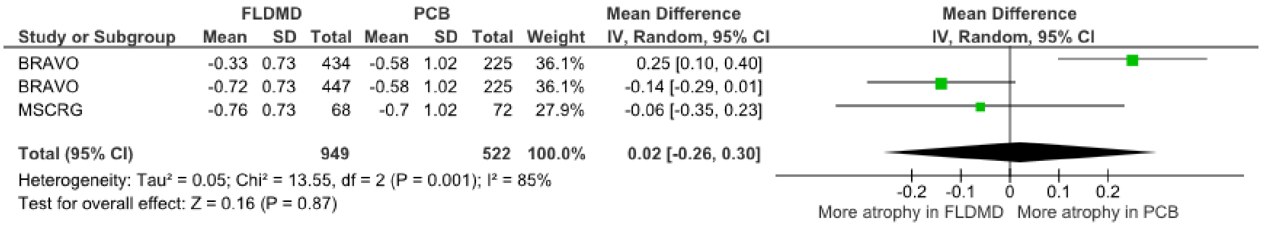

(C)

	SLDMD			PCB				Mean Difference
Study or Subgroup	Mean	SD	Total	Mean	SD	Total	Weight	IV, Random, 95% CI
AFFIRM	-0.8	1.31	580	-0.82	1.5	282	26.4%	0.02 [-0.18, 0.22]
FREEDOMS	-0.84	1.31	357	-1.31	1.5	331	26.4%	0.47 [0.26, 0.68]
FREEDOMS II	-0.86	1.31	264	-1.28	1.5	245	26.1%	0.42 [0.17, 0.67]
Rinaldi	-1.7	1.31	35	-4.6	1.5	35	21.0%	2.90 [2.24, 3.56]
**Total (95% CI)**			**1236**			**893**	**100.0%**	**0.85 [0.21, 1.48]**

Heterogeneity: Tau² = 0.39; Chi² = 68.69, df = 3 (P < 0.00001); I² = 96%
Test for overall effect: Z = 2.62 (P = 0.009)

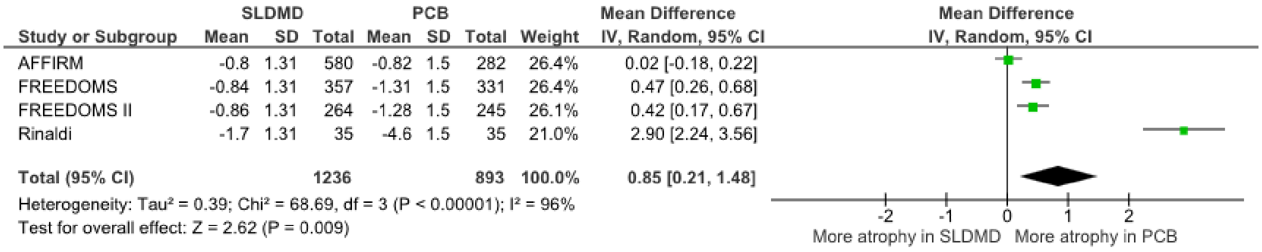

(D)

	FLDMD			PCB				Mean Difference
Study or Subgroup	Mean	SD	Total	Mean	SD	Total	Weight	IV, Random, 95% CI
ALLEGRO	-0.87	0.73	550	-1.3	1.3	556	16.5%	0.43 [0.31, 0.55]
BRAVO	-1.25	0.73	447	-1.14	1.3	225	15.2%	-0.11 [-0.29, 0.07]
BRAVO	-0.83	0.73	434	-1.14	1.3	225	15.2%	0.31 [0.13, 0.49]
COPOLYMER I	-0.6	0.73	14	-1.8	1.3	13	4.2%	1.20 [0.40, 2.00]
DEFINE	-0.64	0.73	151	-0.81	1.3	163	14.0%	0.17 [-0.06, 0.40]
MSCRG	-0.99	0.73	68	-1.22	1.3	72	11.0%	0.23 [-0.12, 0.58]
Rinaldi	-3.7	0.73	50	-4.6	1.3	35	8.3%	0.90 [0.42, 1.38]
TEMSO	-0.39	0.73	358	-0.53	1.3	363	15.8%	0.14 [-0.01, 0.29]
**Total (95% CI)**			**2072**			**1652**	**100.0%**	**0.30 [0.11, 0.48]**

Heterogeneity: Tau² = 0.05; Chi² = 38.36, df = 7 (P < 0.00001); I² = 82%
Test for overall effect: Z = 3.11 (P = 0.002)

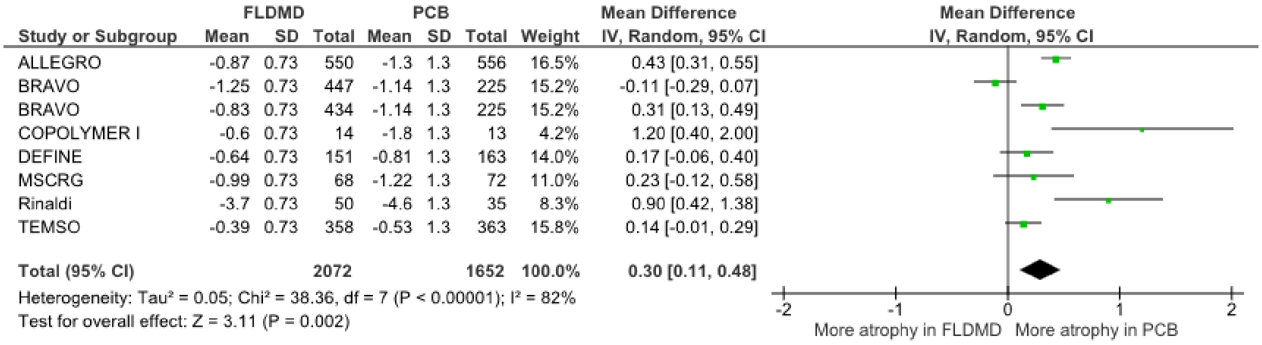

DMD: Disease Modifying Drug; FLDMD: First-line DMD; SLDMD: Second-line DMD; PCB: Placebo; SD: Standard Deviation.
