# Supplementary material for: The Effect of Disease-Modifying Drugs on Brain Atrophy in Relapsing-Remitting Multiple Sclerosis: A Meta-Analysis
Source: PLoS One. 2016 Mar 16;11(3):e0149685. doi: 10.1371/journal.pone.0149685 (PMC4794160; doi:10.1371/journal.pone.0149685)

S4 Fig

Funnel plots of the included studies: FLDMD vs placebo at 12 monts (A), SLDMD vs placebo at 12 monts (B), FLDMD vs SLDMD at 12 months (C), FLDMD vs placebo at 24 monts (D), SLDMD vs placebo at 24 monts (E) and FLDMD vs SLDMD at 24 months (F).

(A)

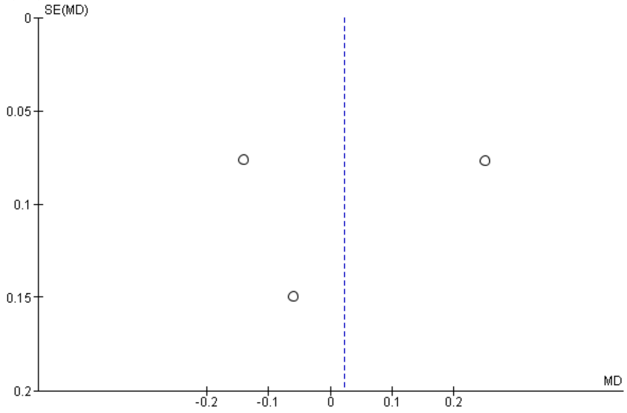

(B)

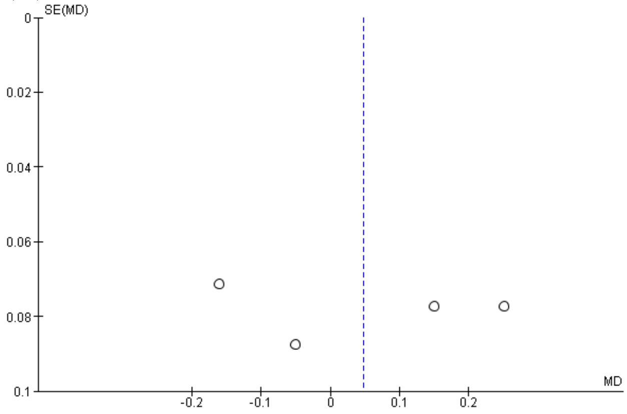

(C)

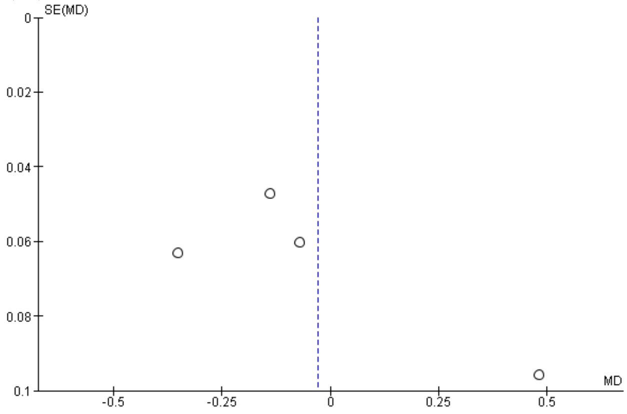

(D)

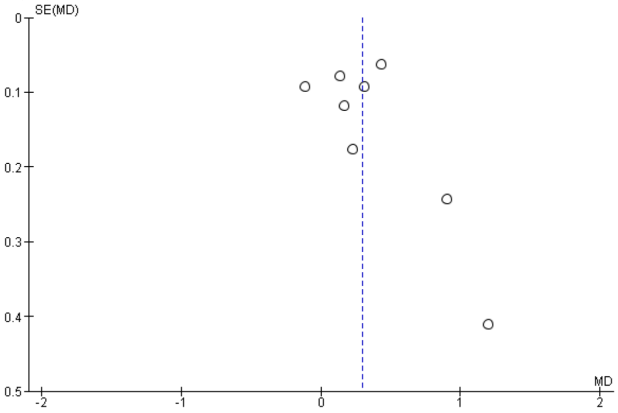

(E)

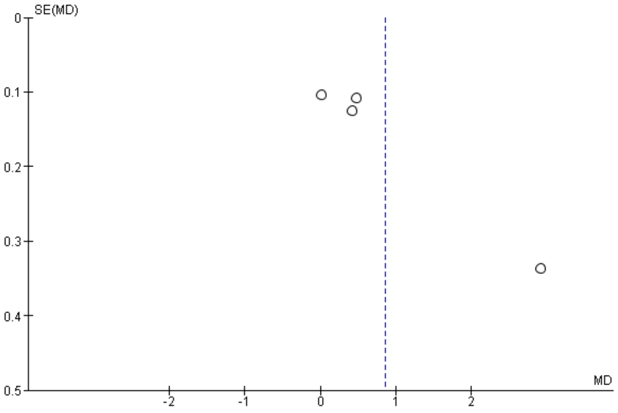

(F)

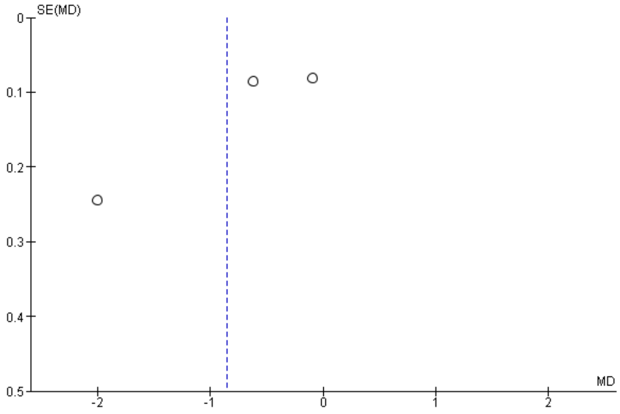

Supplement: S4 Fig — Funnel plots of the included studies: FLDMD vs placebo at 12 monts (A), SLDMD vs placebo at 12 monts (B), FLDMD vs SLDMD at 12 months (C), FLDMD vs placebo at 24 monts (D), SLDMD vs placebo at 24 monts (E) and FLDMD vs SLDMD at 24 months (F). (PDF) [file pone.0149685.s005.pdf]
