# Supplementary material for: The Effect of Disease-Modifying Drugs on Brain Atrophy in Relapsing-Remitting Multiple Sclerosis: A Meta-Analysis
Source: PLoS One. 2016 Mar 16;11(3):e0149685. doi: 10.1371/journal.pone.0149685 (PMC4794160; doi:10.1371/journal.pone.0149685)
Supplement: S1 Table — (PDF) [file pone.0149685.s006.pdf]

S1 Table

Baseline characteristics of included trials.

Study	Year	Technique	Randomized	Type of DMD	DMD	Number of patients	Sex ratio, f/m	Age, years mean	Age, years SD	EDSS, no mean	EDSS, no SD	ARR, no mean	ARR, no SD	Duration, years mean	Duration, years SD
BRAVO [1]	2014	SIENA	yes	FLDMD	IFN-B1a	447	2.19	38.20	9.50	2.60	1.20	1.30	0.60	7.00	5.90
				FLDMD	LAQ 0,6mg	434	1.86	37.00	9.30	2.70	1.30	1.30	0.60	6.60	6.00
				Placebo	Placebo	450	2.48	37.50	9.50	2.70	1.20	1.30	0.60	6.90	6.60
FREEDOMS II [2]	2014	SIENA	yes	SLDMD	FIN 1,25mg	370	3.16	40.90	8.90	2.50	1.30	1.50	1.00	10.80	8.20
				SLDMD	FIN 0,5mg	358	3.31	40.60	8.40	2.40	1.30	1.40	0.90	10.40	8.00
				Placebo	Placebo	355	4.30	40.10	8.40	2.40	1.30	1.50	0.90	10.60	7.90
SELECT [3]	2013	SIENA	yes	SLDMD	DAC 150mg	208	2.03	35.30	8.90	2.80	1.20	1.40	0.70	3.00	NA
				SLDMD	DAC 300mg	209	1.78	35.20	8.70	2.70	1.20	1.30	0.70	3.00	NA
				Placebo	Placebo	204	1.70	36.60	9.00	2.70	1.20	1.30	0.60	2.00	NA
Vidal-Jordana [4]	2013	SIENA	no	SLDMD	NTZ 300mg	39	2.55	34.95	8.75	4.00	NA	NA	NA	10.98	NA
CARE-MS I [5]	2012	BPF	yes	FLDMD	IFN-B1a	187	1.86	33.20	8.50	2.00	0.80	1.80	0.80	2.00	1.30
				SLDMD	ALM 12mg	376	1.86	33.00	8.00	2.00	0.80	1.80	0.80	2.10	1.40
CARE-MS II [6]	2012	BPF	yes	FLDMD	IFN-B1a	202	1.86	35.80	8.77	2.70	1.21	1.50	0.75	4.70	2.86
				SLDMD	ALM 12mg	426	1.94	34.80	8.36	2.70	1.26	1.70	0.86	4.50	2.68
				SLDMD	ALM 24mg	170	2.45	35.10	8.40	2.70	1.17	1.60	0.86	4.30	2.77
DEFINE [7,8]	2012	SIENA	yes	FLDMD	BG-12 480mg	176	3.35	38.30	9.30	2.30	1.20	1.30	0.60	8.70	7.10
				FLDMD	BG-12 720mg	184	3.17	38.50	8.60	2.30	1.20	1.30	0.60	8.00	6.00
				Placebo	Placebo	180	3.55	38.30	9.20	2.50	1.20	1.30	0.70	8.60	6.60
ALLEGRO [9]	2012	SIENA	yes	FLDMD	LAQ 0,6mg	550	2.46	38.90	9.20	2.60	1.30	1.20	0.70	8.70	6.90
				Placebo	Placebo	556	1.96	38.50	9.10	2.60	1.30	1.30	0.70	8.70	6.70
Portaccio [10]	2012	SIENA	no	FLDMD	IFN-B	14	3.67	39.00	9.20	1.30	0.40	1.10	0.30	8.60	3.80
				SLDMD	NTZ	12	3.00	37.30	11.90	3.50	1.60	2.30	0.60	8.10	4.70
Rinaldi [11]	2012	CTh	no	FLDMD	IFN-B1 or AG	50	2.21	36.00	8.20	2.00	1.00	0.50	0.50	5.00	2.20
				SLDMD	NTZ	35	1.50	32.60	7.30	3.20	1.20	2.30	1.00	8.70	4.90
				Placebo	Not treated	35	2.50	39.10	11.20	1.30	0.80	1.00	0.90	6.00	4.80
Ruocco [12]	2012	BPFV	no	FLDMD	IFN-B1 or AG	54	2.18	30.00	9.60	2.60	1.20	1.40	1.00	10.00	5.80
Magraner [13]	2012	SIENA	no	SLDMD	NTZ	18	3.50	31.60	9.00	3.10	0.90	2.00	1.10	7.20	5.70
IMPROVE [14,15]	2012	SIENA	yes	FLDMD	IFN-B1a 44µg	120	NA	NA	NA	NA	NA	NA	NA	NA	NA
				Placebo	Placebo	60	NA	NA	NA	NA	NA	NA	NA	NA	NA
TEMSO [16]	2011	BPF	yes	FLDMD	TER 7mg	366	2.30	37.40	9.00	2.68	1.34	1.40	0.70	8.80	6.80
				FLDMD	TER 14mg	359	2.45	37.80	8.20	2.67	1.24	1.30	0.70	8.70	6.70
				Placebo	Placebo	363	3.13	38.40	9.00	2.68	1.34	1.40	0.70	8.60	7.10
FORTE [17]	2011	SIENA	yes	FLDMD	AG 20mg	586	2.55	36.30	9.00	2.20	1.20	1.50	0.70	3.00	4.00
				FLDMD	AG 40mg	569	2.51	36.30	9.00	2.10	1.10	1.40	0.70	3.30	4.80
QUALICORE [18]	2010	BPF	no	FLDMD	IFN-B1a	28	2.50	32.50	NA	1.70	NA	1.70	NA	2.50	NA
SENTINEL [19,20]	2010	BPF	yes	FLDMD	IFN-B1a 30µg	582	2.60	39.10	7.60	2.50	1.10	1.49	0.72	8.00	NA
Van den Elskamp [21]	2010	SIENA	no	Placebo	oral IFN-B1a or Placebo	135	2.65	35.80	8.60	2.00	NA	NA	NA	6.60	5.50
FREEDOMS [22]	2010	SIENA	yes	SLDMD	FIN 1,25mg	429	2.21	37.40	8.90	2.40	1.40	1.50	0.80	8.40	6.90
				SLDMD	FIN 0,5mg	425	2.29	36.60	8.80	2.30	1.30	1.50	0.80	8.00	6.60
				Placebo	Placebo	418	2.48	37.20	8.60	2.50	1.30	1.40	0.70	8.10	6.40
TRANSFORMS [23]	2010	SIENA	yes	FLDMD	IFN-B1a 30µg	435	2.11	36.00	8.30	2.19	1.26	1.50	0.80	7.40	6.30
				SLDMD	FIN 1,25mg	426	2.21	35.80	8.40	2.21	1.31	1.50	0.90	7.30	6.00
				SLDMD	FIN 0,5mg	431	1.89	36.70	8.80	2.24	1.33	1.50	1.20	7.50	6.20
BEYOND [24]	2009	SIENA	yes	FLDMD	AG 20mg	448	2.13	35.20	NA	2.28	NA	1.60	NA	5.10	NA
				FLDMD	IFN-B1b 500µg	899	2.33	35.90	NA	2.33	NA	1.60	NA	5.40	NA
				FLDMD	IFN-B1b 250µg	897	2.33	35.80	NA	2.35	NA	1.60	NA	5.30	NA
ASA [25]	2009	SIENA	yes	FLDMD	IFN-B1a 30µg	60	4.00	28.90	7.10	1.80	0.90	1.80	0.80	4.40	4.20
CAMMS223 [26]	2008	SBV	yes	FLDMD	IFN-B1a	111	1.78	32.80	8.80	1.90	0.83	NA	NA	1.40	NA
				SLDMD	ALM 12mg	112	1.80	31.90	8.00	1.90	0.74	NA	NA	1.30	NA
				SLDMD	ALM 24mg	110	1.82	32.20	8.80	2.00	0.73	NA	NA	1.20	NA
REGARD [27]	2008	SIENA	yes	FLDMD	AG 20mg	378	2.57	36.80	9.50	2.33	1.31	NA	NA	6.55	7.10
				FLDMD	IFN-B1a 44µg	386	2.23	36.70	9.80	2.35	1.28	NA	NA	5.93	6.25
CORAL [28]	2008	CBV	yes	Placebo	Placebo	548	2.70	36.00	7.00	2.00	NA	NA	NA	7.80	6.20
Zivadinov [29]	2007	BPF	no	FLDMD	IFN-B1a	26	1.60	45.00	NA	2.00	NA	NA	NA	13.00	NA
				Placebo	Placebo	28	3.00	47.00	NA	1.00	NA	NA	NA	12.50	NA
AFFIRM [30,31]	2007	BPF	yes	SLDMD	NTZ 300mg	627	2.57	35.60	8.50	2.30	1.20	NA	NA	5.00	NA
				Placebo	Placebo	315	2.03	36.70	7.80	2.30	1.20	NA	NA	6.00	NA
FTY720 D2201 [32]	2006	SIENA	yes	SLDMD	FIN 1,25mg	93	3.00	38.00	NA	2.70	NA	1.30	NA	8.60	NA
				SLDMD	FIN 5mg	92	2.45	38.30	NA	2.50	NA	1.30	NA	9.50	NA
				Placebo	Placebo	92	1.94	37.10	NA	2.60	NA	1.20	NA	8.40	NA
Hardmeier [33]	2005	BPF	no	FLDMD	IFNB1a 30µg or 60µg	386	NA	37.00	7.40	3.50	NA	NA	NA	7.30	5.70
Frank [34]	2004	BV	no	FLDMD	IFN-B1b	30	NA	NA	NA	2.80	2.10	NA	NA	NA	NA
PRISMS [35]	2003	BPV	yes	FLDMD	IFN-B1a 22µg or 44µg	13	2.33	32.00	NA	2.00	NA	NA	NA	4.00	NA
				Placebo	Placebo	7	2.33	32.00	NA	2.00	NA	NA	NA	4.00	NA
Paolillo [36,37]	2002	SBV	yes	FLDMD	IFN-B1a 33µg	57	2.33	30.20	7.70	2.00	1.20	1.60	1.20	5.20	2.80
EUROPEAN / CANADIAN [38,39]	2001	CBV	yes	FLDMD	AG 20mg	113	3.35	34.40	7.40	2.30	1.10	NA	NA	8.00	5.55
				Placebo	Placebo	114	2.68	34.00	7.60	2.40	1.20	NA	NA	8.30	5.58
MSCRG [40,41]	2000	BPF	yes	FLDMD	IFN-B1a	68	NA	36.50	7.20	2.32	0.79	NA	NA	6.60	5.80
				Placebo	Placebo	72	NA	36.40	7.10	2.38	0.91	NA	NA	6.20	5.50
COPOLYMER I [42,43]	2000	BPV	yes	FLDMD	AG 20mg	14	NA	37.10	NA	3.00	NA	NA	NA	5.20	NA
				Placebo	Placebo	13	NA	34.40	NA	2.40	NA	NA	NA	4.20	NA

EDSS: Expended Disability Status Scale; ARR: Annual Relapse Rate; Duration: disease duration between first relapse or diagnostic; SD: Standard Deviation; SIENA: Structural Image Evaluation, using Normalisation, of Atrophy; BPF: Brain Parenchymal Fraction; CTh: Cortical Thickness; BPFV: Brain Parenchymal Fraction Volume; SBV: Supratentorial Brain Volume; CBV: Central Brain Volume; BV: Brain Volume; BPV: Brain Parenchymal Volume; DMD: Disease Modifying Drug; FLDMD: First-line DMD; SLDMD, Second-line DMD: GA, Glatiramer Acetate; IFN: Interferon; TER: Terifluomide; LAQ: Laquinimod; BG-12: dimethyl fumarate; NTZ: Natalizumab; FIN: Fingolimod; ALM: Alemtuzumab; DAC: Daclizumab; NA: Not Available.

1. Vollmer TL, Sorensen PS, Selmaj K, Zipp F, Havrdova E, Cohen JA, et al. A randomized placebo-controlled phase III trial of oral laquinimod for multiple sclerosis. J Neurol. 2014;261: 773–783. PMID: 24535134

2. Calabresi PA, Radue E-W, Goodin D, Jeffery D, Rammohan KW, Reder AT, et al. Safety and efficacy of fingolimod in patients with relapsing-remitting multiple sclerosis (FREEDOMS II): a double-blind, randomised, placebo-controlled, phase 3 trial. Lancet Neurol. 2014;13: 545–556. PMID: 24685276

3. Gold R, Giovannoni G, Selmaj K, Havrdova E, Montalban X, Radue E-W, et al. Daclizumab high-yield process in relapsing-remitting multiple sclerosis (SELECT): a randomised, double-blind, placebo-controlled trial. Lancet. 2013;381: 2167–2175. PMID: 23562009

4. Vidal-Jordana A, Sastre-Garriga J, Pérez-Miralles F, Tur C, Tintoré M, Horga A, et al. Early brain pseudoatrophy while on natalizumab therapy is due to white matter volume changes. Mult Scler. 2013;19: 1175–1181. PMID: 23319072

5. Cohen JA, Coles AJ, Arnold DL, Confavreux C, Fox EJ, Hartung H-P, et al. Alemtuzumab versus interferon beta 1a as first-line treatment for patients with relapsing-remitting multiple sclerosis: a randomised controlled phase 3 trial. Lancet. 2012;380: 1819–1828. PMID: 23122652

6. Coles AJ, Twyman CL, Arnold DL, Cohen JA, Confavreux C, Fox EJ, et al. Alemtuzumab for patients with relapsing multiple sclerosis after disease-modifying therapy: a randomised controlled phase 3 trial. Lancet. 2012;380: 1829–1839. PMID: 23122650

7. Gold R, Kappos L, Arnold DL, Bar-Or A, Giovannoni G, Selmaj K, et al. Placebo-controlled phase 3 study of oral BG-12 for relapsing multiple sclerosis. N Engl J Med. 2012;367: 1098–1107. PMID: 22992073

8. Arnold DL, Gold R, Kappos L, Bar-Or A, Giovannoni G, Selmaj K, et al. Effects of BG-12 (Dimethyl Fumarate) on Magnetic Resonance Imaging and Magnetization Transfer Ratio Outcomes in Relapsing–Remitting Multiple Sclerosis: Findings from the Phase 3 DEFINE Study. 2012 Oct; 28th Congress of the European Committee for Treatment and Research in Multiple Sclerosis, Lyon, France.

9. Comi G, Jeffery D, Kappos L, Montalban X, Boyko A, Rocca MA, et al. Placebo-controlled trial of oral laquinimod for multiple sclerosis. N Engl J Med. 2012;366: 1000–1009. PMID: 22417253

10. Portaccio E, Stromillo ML, Goretti B, Hakiki B, Giorgio A, Rossi F, et al. Natalizumab may reduce cognitive changes and brain atrophy rate in relapsing-remitting multiple sclerosis: a prospective, non-randomized pilot study. Eur J Neurol. 2012; PMID: 23057658

11. Rinaldi F, Calabrese M, Seppi D, Puthenparampil M, Perini P, Gallo P. Natalizumab strongly suppresses cortical pathology in relapsing-remitting multiple sclerosis. Mult Scler. 2012;18: 1760–1767. PMID: 22570359

12. Ruocco HH, Brandão CO, Farias AS, Oliveira C, Oliveira EC, Cendes F, et al. Quantitative MRI and cerebrospinal fluid inflammatory mediators in Brazilian patients with relapsing-remitting multiple sclerosis before and after treatment with immunomodulators: a longitudinal study. Neuroimmunomodulation. 2012;19: 277–282. PMID: 22472803

13. Magraner M, Coret F, Casanova B. The relationship between inflammatory activity and brain atrophy in natalizumab treated patients. Eur J Radiol. 2012;81: 3485–3490. PMID: 22391507

14. De Stefano N, Sormani MP, Stubinski B, Blevins G, Drulovic JS, Issard D, et al. Efficacy and safety of subcutaneous interferon β-1a in relapsing-remitting multiple sclerosis: further outcomes from the IMPROVE study. J Neurol Sci. 2012;312: 97–101. PMID: 21880336

15. De Stefano N, Curtin F, Stubinski B, Blevins G, Drulovic J, Issard D, et al. Rapid benefits of a new formulation of subcutaneous interferon beta-1a in relapsing-remitting multiple sclerosis. Mult Scler. 2010;16: 888–892. PMID: 20200197

16. O'Connor P, Wolinsky JS, Confavreux C, Comi G, Kappos L, Olsson TP, et al. Randomized trial of oral teriflunomide for relapsing multiple sclerosis. N Engl J Med. 2011;365: 1293–1303. PMID: 21991951

17. Comi G, Cohen JA, Arnold DL, Wynn D, Filippi M. Phase III dose-comparison study of glatiramer acetate for multiple sclerosis. Ann Neurol. 2011;69: 75–82. PMID: 21280077

18. Cohen M, Lebrun C, Aufauvre D, Chanalet S, Filleau-Bertogliatti C, Camu W, et al. [Longitudinal study of health related quality of life in multiple sclerosis: correlation with MRI parameters]. Rev Neurol (Paris). 2010;166: 894–900. PMID: 20728910

19. Radue E-W, Stuart WH, Calabresi PA, Confavreux C, Galetta SL, Rudick RA, et al. Natalizumab plus interferon beta-1a reduces lesion formation in relapsing multiple sclerosis. J Neurol Sci. 2010;292: 28–35. PMID: 20236661

20. Rudick RA, Stuart WH, Calabresi PA, Confavreux C, Galetta SL, Radue E-W, et al. Natalizumab plus interferon beta-1a for relapsing multiple sclerosis. N Engl J Med. 2006;354: 911–923. PMID: 16510745

21. van den Elskamp IJ, Boden B, Dattola V, Knol DL, Filippi M, Kappos L, et al. Cerebral atrophy as outcome measure in short-term phase 2 clinical trials in multiple sclerosis. Neuroradiology. 2010;52: 875–881. PMID: 20049424

22. Kappos L, Radue E-W, O'Connor P, Polman C, Hohlfeld R, Calabresi P, et al. A placebo-controlled trial of oral fingolimod in relapsing multiple sclerosis. N Engl J Med. 2010;362: 387–401. PMID: 20089952

23. Cohen JA, Barkhof F, Comi G, Hartung H-P, Khatri BO, Montalban X, et al. Oral fingolimod or intramuscular interferon for relapsing multiple sclerosis. N Engl J Med. 2010;362: 402–415. PMID: 20089954

24. O'Connor P, Filippi M, Arnason B, Comi G, Cook S, Goodin D, et al. 250 microg or 500 microg interferon beta-1b versus 20 mg glatiramer acetate in relapsing-remitting multiple sclerosis: a prospective, randomised, multicentre study. Lancet Neurol. 2009;8: 889–897. PMID: 19729344

25. Havrdova E, Zivadinov R, Krasensky J, Dwyer MG, Novakova I, Dolezal O, et al. Randomized study of interferon beta-1a, low-dose azathioprine, and low-dose corticosteroids in multiple sclerosis. Mult Scler. 2009;15: 965–976. PMID: 19465443

26. CAMMS223 Trial Investigators, Coles AJ, Compston DAS, Selmaj KW, Lake SL, Moran S, et al. Alemtuzumab vs. interferon beta-1a in early multiple sclerosis. N Engl J Med. 2008;359: 1786–1801. PMID: 18946064

27. Mikol DD, Barkhof F, Chang P, Coyle PK, Jeffery DR, Schwid SR, et al. Comparison of subcutaneous interferon beta-1a with glatiramer acetate in patients with relapsing multiple sclerosis (the REbif vs Glatiramer Acetate in Relapsing MS Disease [REGARD] study): a multicentre, randomised, parallel, open-label trial. Lancet Neurol. 2008;7: 903–914. PMID: 18789766

28. Mesaros S, Rocca MA, Sormani MP, Charil A, Comi G, Filippi M. Clinical and conventional MRI predictors of disability and brain atrophy accumulation in RRMS. A large scale, short-term follow-up study. J Neurol. 2008;255: 1378–1383. PMID: 18584233

29. Zivadinov R, Locatelli L, Cookfair D, Srinivasaraghavan B, Bertolotto A, Ukmar M, et al. Interferon beta-1a slows progression of brain atrophy in relapsing-remitting multiple sclerosis predominantly by reducing gray matter atrophy. Mult Scler. 2007;13: 490–501. PMID: 17463072

30. Miller DH, Soon D, Fernando KT, MacManus DG, Barker GJ, Yousry TA, et al. MRI outcomes in a placebo-controlled trial of natalizumab in relapsing MS. Neurology. 2007;68: 1390–1401. PMID: 17452584

31. Polman CH, O'Connor PW, Havrdova E, Hutchinson M, Kappos L, Miller DH, et al. A randomized, placebo-controlled trial of natalizumab for relapsing multiple sclerosis. N Engl J Med. 2006;354: 899–910. PMID: 16510744

32. Kappos L, Antel J, Comi G, Montalban X, O'Connor P, Polman CH, et al. Oral fingolimod (FTY720) for relapsing multiple sclerosis. N Engl J Med. 2006;355: 1124–1140. PMID: 16971719

33. Hardmeier M, Wagenpfeil S, Freitag P, Fisher E, Rudick RA, Kooijmans M, et al. Rate of brain atrophy in relapsing MS decreases during treatment with IFNbeta-1a. Neurology. 2005;64: 236–240. PMID: 15668419

34. Frank JA, Richert N, Bash C, Stone L, Calabresi PA, Lewis B, et al. Interferon-beta-1b slows progression of atrophy in RRMS: Three-year follow-up in NAb- and NAb+ patients. Neurology. 2004;62: 719–725. PMID: 15007120

35. Turner B, Lin X, Calmon G, Roberts N, Blumhardt LD. Cerebral atrophy and disability in relapsing-remitting and secondary progressive multiple sclerosis over four years. Mult Scler. 2003;9: 21–27. PMID: 12617263

36. Paolillo A, Pozzilli C, Giugni E, Tomassini V, Gasperini C, Fiorelli M, et al. A 6-year clinical and MRI follow-up study of patients with relapsing-remitting multiple sclerosis treated with Interferon-beta. Eur J Neurol. 2002;9: 645–655. PMID: 12453081

37. Pozzilli C, Bastianello S, Koudriavtseva T, Gasperini C, Bozzao A, Millefiorini E, et al. Magnetic resonance imaging changes with recombinant human interferon-beta-1a: a short term study in relapsing-remitting multiple sclerosis. J Neurol Neurosurg Psychiatr. 1996;61: 251–258. PMID: 8795595

38. Rovaris M, Comi G, Rocca MA, Wolinsky JS, Filippi M. Short-term brain volume change in relapsing-remitting multiple sclerosis: effect of glatiramer acetate and implications. Brain. 2001;124: 1803–1812. PMID: 11522582

39. Comi G, Filippi M, Wolinsky JS. European/Canadian multicenter, double-blind, randomized, placebo-controlled study of the effects of glatiramer acetate on magnetic resonance imaging--measured disease activity and burden in patients with relapsing multiple sclerosis. European/Canadian Glatiramer Acetate Study Group. Ann Neurol. 2001;49: 290–297. PMID: 11261502

40. Rudick RA, Fisher E, Lee JC, Duda JT, Simon J. Brain atrophy in relapsing multiple sclerosis: relationship to relapses, EDSS, and treatment with interferon beta-1a. Mult Scler. 2000;6: 365–372. PMID: 11212130

41. Jacobs LD, Cookfair DL, Rudick RA, Herndon RM, Richert JR, Salazar AM, et al. Intramuscular interferon beta-1a for disease progression in relapsing multiple sclerosis. The Multiple Sclerosis Collaborative Research Group (MSCRG). Ann Neurol. 1996;39: 285–294. PMID: 8602746

42. Ge Y, Grossman RI, Udupa JK, Wei L, Mannon LJ, Polansky M, et al. Brain atrophy in relapsing-remitting multiple sclerosis and secondary progressive multiple sclerosis: longitudinal quantitative analysis. Radiology. 2000;214: 665–670. PMID: 10715027

43. Johnson KP, Brooks BR, Cohen JA, Ford CC, Goldstein J, Lisak RP, et al. Copolymer 1 reduces relapse rate and improves disability in relapsing-remitting multiple sclerosis: results of a phase III multicenter, double-blind placebo-controlled trial. The Copolymer 1 Multiple Sclerosis Study Group. Neurology. 1995;45: 1268–1276. PMID: 7617181
