# Supplementary material for: The Effect of Disease-Modifying Drugs on Brain Atrophy in Relapsing-Remitting Multiple Sclerosis: A Meta-Analysis
Source: PLoS One. 2016 Mar 16;11(3):e0149685. doi: 10.1371/journal.pone.0149685 (PMC4794160; doi:10.1371/journal.pone.0149685)
Supplement: S1 Text — (PDF) [file pone.0149685.s007.pdf]

S1 Text

MEDLINE search algorithm.

("multiple sclerosis"[MeSH Terms] OR "multiple sclerosis"[All Fields] OR ("multiple"[All Fields] AND "sclerosis"[All Fields])) AND ("atrophy"[MeSH Terms] OR "atrophy"[All Fields]) AND ("glatiramer" [All Fields] OR "acetate" [All Fields] OR "glatiramer acetate"[All Fields] OR "interferon"[All Fields] OR "teriflunomide"[All Fields] OR "dimethyl"[All Fields] OR "fumarate"[All Fields] OR "dimethyl fumarate"[All Fields] OR "BG12"[All Fields] OR "BG-12"[All Fields] OR "laquinimod"[All Fields] OR "natalizumab"[All Fields] OR "fingolimod"[All Fields] OR "alemtuzumab"[All Fields] OR "daclizumab"[All Fields] OR "ocrelizumab"[All Fields]) AND ("1990/01/01"[CRDAT] : "2014/06/01"[CRDAT]).
